# Genome‐Wide Identification and Functional Characterization of New Serotonin N‐Acetyltransferases in Soybean

**DOI:** 10.1002/fsn3.70147

**Published:** 2025-04-06

**Authors:** Jiajia Zhang, Haopeng Li, Hongfei Ma, Wei Yan, Jianbo Zhu

**Affiliations:** ^1^ College of Life Sciences Shihezi University Shihezi China

**Keywords:** leguminosae, melatonin, serotonin N‐acetyltransferase, soybean

## Abstract

Melatonin is recognized as a crucial plant hormone due to its significant role in plant development, growth promotion, enhancing antioxidant activity, and stress resistance. Soybean is one of the most important crops globally. To date, three SNAT genes have been cloned in Arabidopsis and rice; however, only one gene, *GmSNAT1*, has been characterized in soybeans. In this study, members of the GmGNAT family were identified through genome‐wide analysis. Two candidate GmSNAT genes (*GmSNAT3.1* and *GmSNAT3.2*) were selected based on phylogenetic tree analysis and exhibited highly similar protein tertiary structures to ZmSNAT3. Furthermore, the functionality of these two GmSNAT genes was confirmed for synthesizing N‐acetylserotonin or melatonin using serotonin or 5‐methoxytryptamine as substrates in vitro. RNA‐seq data indicated that the transcription levels of *GmSNAT3.1* and *GmSNAT3.2* were downregulated under conditions of dehydration and salt stress. Additionally, SNAT3 genes from other leguminous plants were identified through homology analysis with GmSNAT genes. This study elucidates the role of SNAT genes in soybeans and establishes a foundation for further exploration into the physiological functions of melatonin within legume plants.

## Graphical abstract

Two new GmSNAT genes were confirmed to be able to synthesize N‐acetylserotonin or melatonin using serotonin or 5‐methoxytryptamine as substrates. The transcription levels of GmSNAT3.1 and GmSNAT3.2 were downregulated under conditions of dehydration and salt stress. SNAT3 genes from other leguminous plants were also identified.

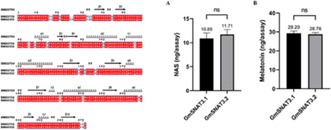

## 1 Introduction

Melatonin, commonly recognized for its role in regulating sleep in animals, also serves a vital function in plants. The biosynthesis pathway of melatonin in plants is generally conserved and comprises four steps involving five key enzymes: tryptophan decarboxylase (TDC), tryptamine 5‐hydroxylase (T5H), serotonin N‐acetyltransferase (SNAT), N‐acetyl‐serotonin methyltransferase (ASMT), and caffeic acid O‐methyltransferase (COMT). Research has identified two distinct pathways for melatonin synthesis in plants, with the subcellular localization of these enzymes influencing the regulation of the synthesis process (Arnao and Hernández‐Ruiz 2020; Gatti et al. 2024). The penultimate step in melatonin biosynthesis within plants is catalyzed by serotonin N‐acetyltransferase (SNAT), which facilitates the conversion of serotonin (5‐hydroxytryptamine) to N‐acetyl‐serotonin or transforms 5‐methoxytryptamine into melatonin. Recent studies have elucidated that SNAT functions as the key rate‐limiting enzyme within this biosynthetic pathway. It belongs to the general control non‐repressible 5 (GCN5)‐related N‐acetyltransferases (GNAT) family, which encompasses activities such as N‐α‐acetylation (NTA) and ε‐lysine acetylation (KA).

Current investigations have successfully cloned *OsSNAT1* (Kang et al. 2013) and *OsSNAT2* (Byeon et al. 2016) from rice and validated their catalytic activity in vitro. Analysis of the amino acid sequences of SNAT proteins reveals that OsSNAT1 and OsSNAT2 share a 39% identity and exhibit a similarity of approximately 60%. Furthermore, phylogenetic analysis indicates that OsSNAT1 and OsSNAT2 are distantly related, suggesting they may have evolved independently from cyanobacteria prior to the endosymbiotic event. The SNAT genes in Arabidopsis have been identified, exhibiting only 27% homology between AtSNAT1 and AtSNAT2 proteins (Lee, Byeon, Back, et al. 2014; Lee et al. 2019). This relationship provides valuable insights into protein structure and function prediction, as well as the evolutionary relationships among these proteins, which are crucial for advancing the field of protein bioinformatics. *OsSNAT3* has also been identified and its overexpression lines showed higher melatonin levels. The SNAT3‐OE lines were more tolerant in cadmium stress (Lee and Back 2024). Recently, *AtSNAT6* was also cloned in Arabidopsis, and its homologous gene, *ZmSNAT3*, was identified in maize (Wang et al. 2022; Guo et al. 2024). The transcription of *ZmSNAT1* and *ZmSNAT3* was responsive to heat and drought stresses.

Soybean ( *Glycine max* (L.) Merr.) is one of the most economically significant crops globally, playing a vital role in supporting various industries. Despite its importance, soybean is highly susceptible to environmental stresses such as drought, salinity, and high temperatures, which severely impact its yield and quality (Huang et al. 2024; Hamza et al. 2024). With the ongoing changes in global climate, enhancing soybean's resilience to environmental stresses has become increasingly urgent. Melatonin, a key regulator of physiological processes with conserved functions across species, plays a critical role in plant development and stress responses (Gao et al. 2023; Zargar et al. 2025). However, research on melatonin biosynthesis genes, particularly those encoding SNAT, remains limited in soybeans compared to Arabidopsis and maize. To date, only GmSNAT1 has been shown to exhibit serotonin N‐acetyltransferase activity (Kumar et al. 2022). The successful cloning of melatonin biosynthesis genes in other crops has provided a solid foundation for advancing similar research in soybeans. The cloning of SNAT genes offers a promising approach to enhance yield and stress tolerance in soybean, while also providing a valuable tool for elucidating the specific physiological functions of endogenous melatonin in this important crop.

In this study, we identified the *GmSNAT3.1* and *GmSNAT3.2* genes in soybeans and enzymatically characterized the recombinant SNAT proteins derived from them. Additionally, we performed a comparative analysis of the tertiary structures of GmSNATs and ZmSNAT3. The transcription levels of *GmSNAT3.1* and *GmSNAT3.2* in response to dehydration and salt stress were investigated by the public RNA‐seq dataset. Furthermore, we identified SNAT3 homologs in several other legume species, shedding light on the evolutionary conservation and functional significance of these genes in the Leguminosae family.

## 2 Results

### 2.1 Identification of the GNAT Gene Family in Soybean

A comprehensive analysis identified 112 members of the GNAT (GCN5‐related N‐acetyltransferase) family in soybeans ( *Glycine max* ). Among these, 34 proteins were found to contain chloroplast transit peptides (cTP), suggesting their potential localization in chloroplasts (Table 1). The molecular weights of the GNAT proteins span a broad range, from 14.8 to 146.5 kDa, while their isoelectric points (pI) vary between 5.29 and 10.53, reflecting their diverse biochemical properties. Additionally, the protein lengths exhibit significant variation, ranging from 133 to 1319 amino acids (Data S1). This extensive diversity highlights the functional versatility and complexity of the GNAT family in soybeans (Table 1).

**TABLE 1:** The prediction of chloroplast transit peptide of SNAT3 protein in some legume species.

Name	Transcript ID	Length	Score cTP	cTP length
GmSNAT3.1	KRH23754	271	0.547 Y	51
GmSNAT3.2	KRH44932	273	0.540 Y	53
PvSNAT3	ESW03293	281	0.565 Y	49
VfSNAT3	2 g227160.1	279	0.526 Y	51
AhSNAT3.1	Ah03g122100.1	274	0.555 Y	32
AhSNAT3.2	Ah13g157600.1	274	0.553 Y	32
VuSNAT3	Vigun11g224400.1	281	0.570 Y	49
CcSNAT3	C.cajan_21792.t	279	0.520 Y	51
MtSNAT3	RHN59586.1	284	0.518 Y	53
PsSNAT3	Psat7g147160.1	278	0.516 Y	54

Abbreviations: Ah, *Arachis hypogaea* ; Cc, *Cajanus cajan* ; Mt, *Medicago truncatula* ; Ps, *Pisum sativum* ; Pv, *Phaseolus vulgaris* ; Vf, *Vicia faba* ; Vu, *Vigna unguiculata*.

### 2.2 Chromosome Position and Domain Analysis of GNAT Gene Family Members

**FIGURE 1:**
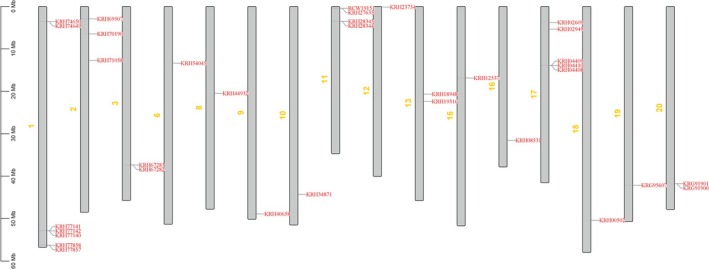
The chromosomal distribution of 34 GmGNAT genes in soybean.

**FIGURE 2:**
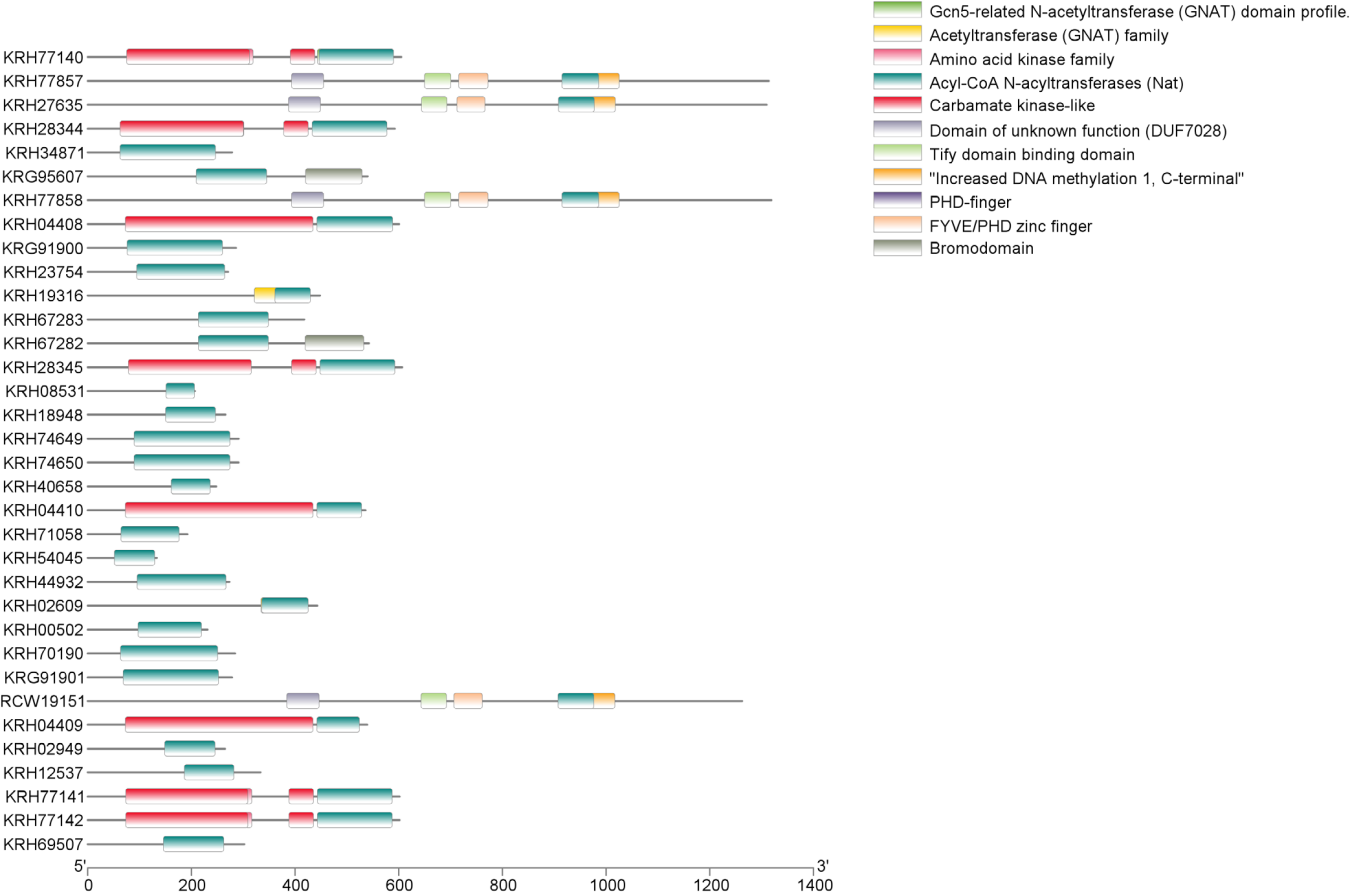
Conserved domain analysis of GmGNATs proteins.

Chromosomal localization analysis has demonstrated that *GmGNAT* genes are distributed across 16 out of the 20 soybean chromosomes (Figure 1). Conserved domain analysis revealed that all GmGNAT proteins contain the Acyl‐CoA N‐acyltransferases domain at their C‐terminus, a hallmark of this protein family. Interestingly, some of the longer GmGNAT proteins were found to possess additional functional domains, including the Carbamate kinase‐like domain, Tify domain binding domain, and FYVE/PHD zinc finger domain, which are known to play pivotal roles in chromatin regulation and transcriptional processes (Figure 2). The presence of these domains suggests that these proteins may have evolved multifunctional roles beyond their canonical N‐acyltransferase activity, potentially integrating metabolic and regulatory functions in cellular processes.

### 2.3 Candidate GmSNAT Screening and Sequence Analysis

**FIGURE 3:**
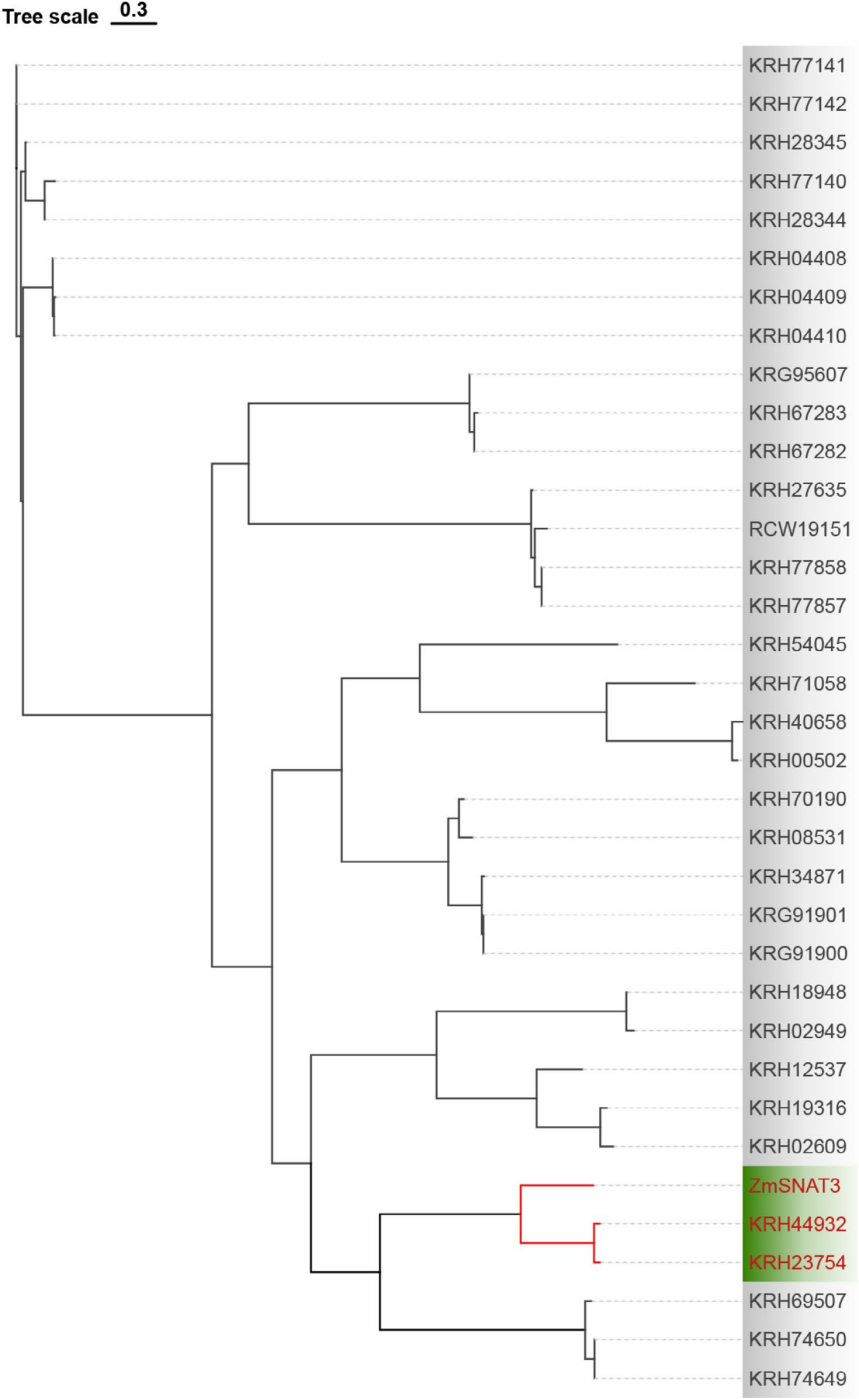
The phylogenetic tree of GmGNATs and ZmSNAT3.

**FIGURE 4:**
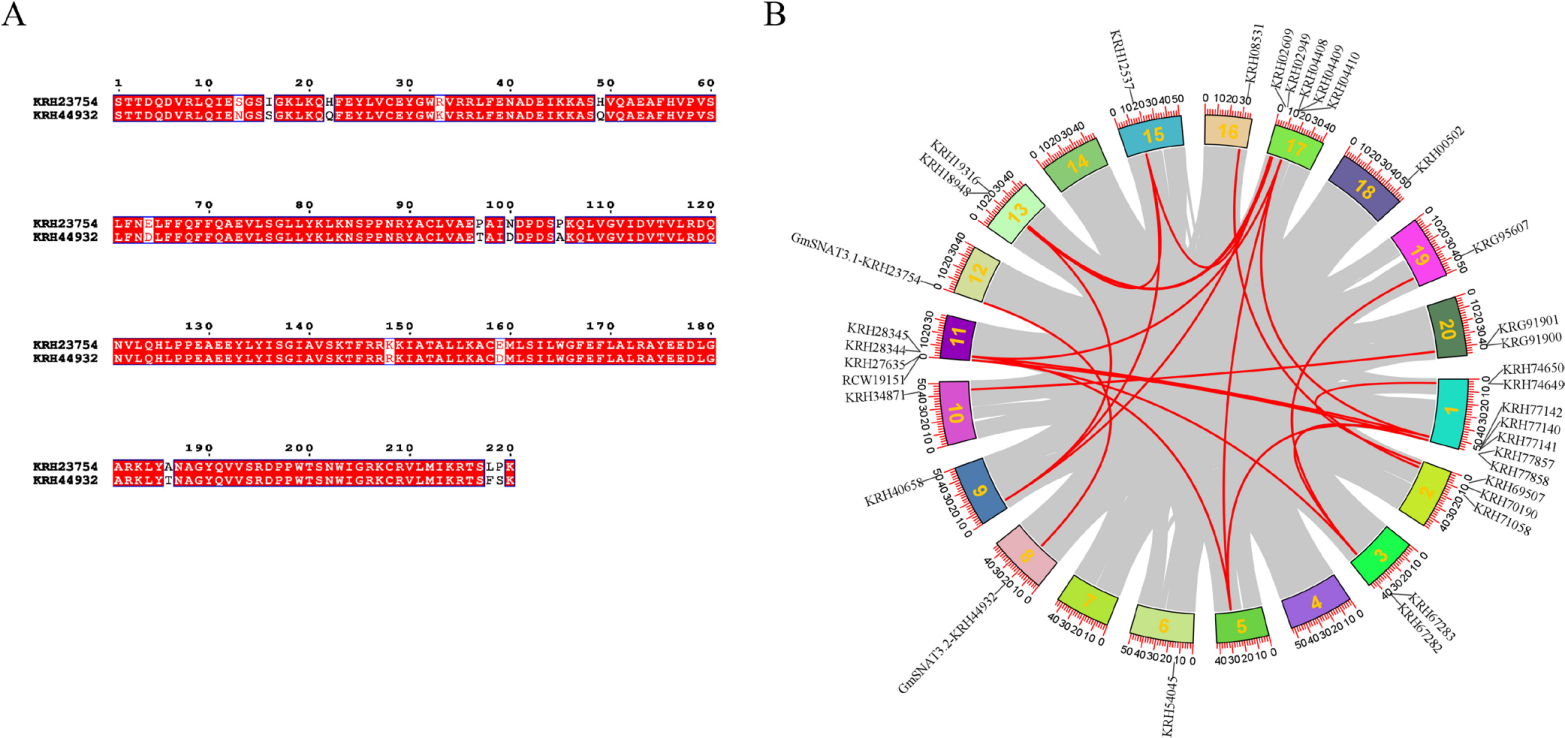
The sequence alignment and the collinearity analysis of GmSNAT candidates. (A) The alignment of GmSNAT candidate genes. (B) Collinearity analysis of GmGNAT genes. The gray lines in the background represent collinear modules in the soybean genome. The red lines indicate GmGNAT gene pairs with collinearity.

The phylogenetic analysis incorporating the newly cloned maize ZmSNAT3 gene indicates that two soybean genes, KRH23754 and KRH44932, exhibit a high degree of homology with ZmSNAT3 (Figure 3). The protein sequences of KRH23754 and KRH44932 (excluding the chloroplast transit peptide, cTP) share an identity of 93.64% (Figure 4A). Moreover, these genes are located within syntenic blocks, suggesting that their functions are conserved (Figure 4B). Based on these findings, KRH23754 and KRH44932 were selected for further validation and have been designated as GmSNAT3.1 and GmSNAT3.2, respectively.

Phylogenetic analysis, incorporating the newly cloned maize *ZmSNAT3* gene, revealed that two soybean gene transcripts, KRH23754 and KRH44932, exhibit a high degree of homology with ZmSNAT3 (Figure 3), despite the absence of synteny conservation between these genomic regions (Figure S1). The protein sequences of KRH2375 and KRH44932 (excluding the chloroplast transit peptide, cTP) share 93.64% identity (Figure 4A), indicating strong evolutionary conservation. Furthermore, these genes are located within syntenic genomic blocks, further supporting the conservation of their functional roles (Figure 4B). Based on these findings, KRH23754 and KRH44932 were selected as the candidates for further functional validation and have been designated as GmSNAT3.1 and GmSNAT3.2, respectively.

### 2.4 Comparison of Protein Tertiary Structures

**FIGURE 5:**
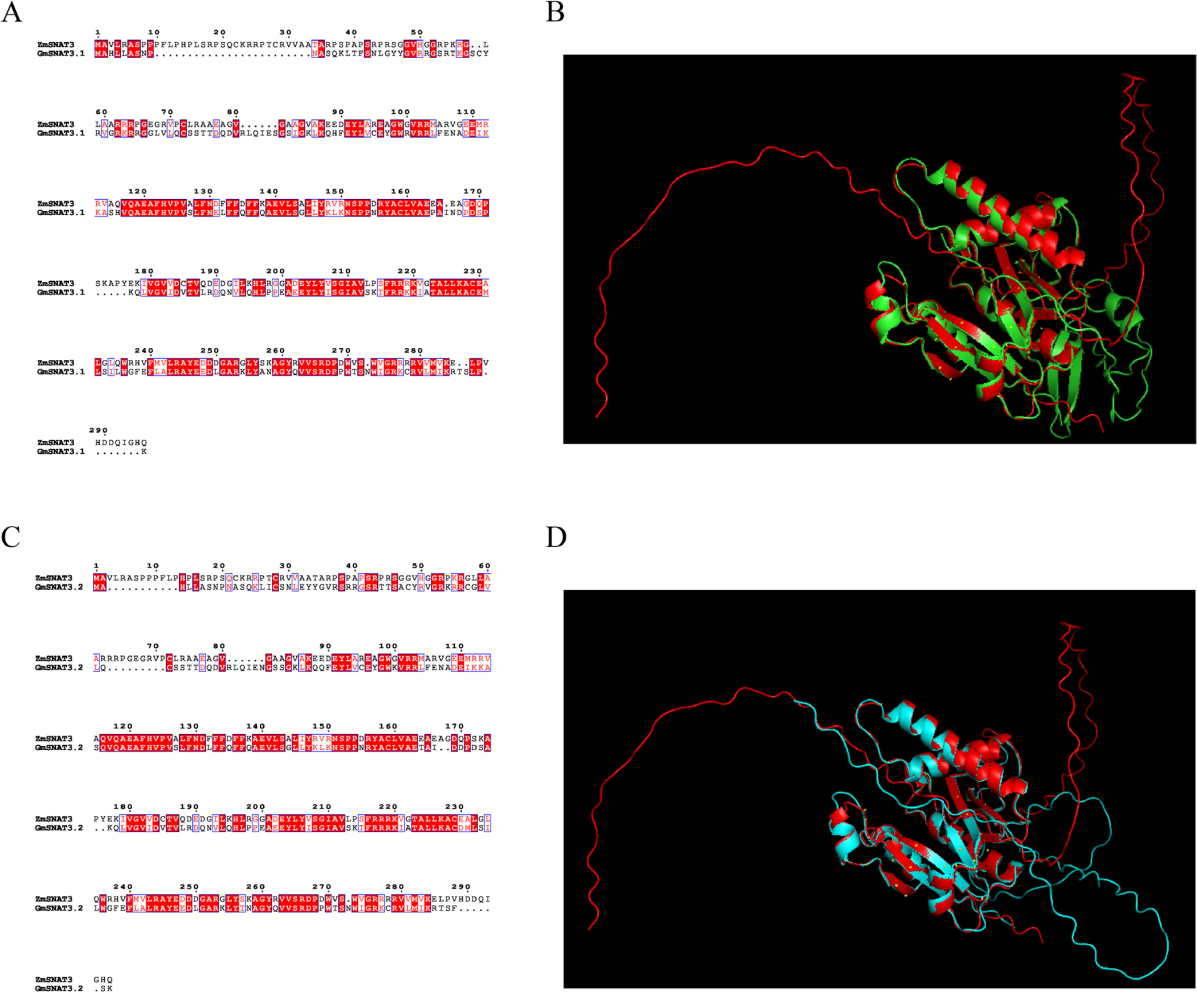
Comparison of protein sequences and tertiary structures. (A) The amino acids sequence alignment of GmSNAT3.1 and ZmSNAT3. (B) The overall alignment of ZmSNAT3 (red) and GmSNAT3.1 (green) proteins. (C) The amino acids sequence alignment of GmSNAT3.2 and ZmSNAT3. (D) The overall alignment of ZmSNAT3 (red) and GmSNAT3.2 (cyan) proteins.

GmSNAT3.1 and GmSNAT3.2 displayed 56.68% and 58.60% sequence similarity to ZmSNAT3 at the protein level, respectively (Figure 5A,C). Despite these sequence variations, their tertiary structures exhibited a high degree of conservation, as evidenced by root mean square deviation (RMSD) values of 0.412 and 0.562 for structural alignment (Figure 5B,D). This structural similarity was particularly pronounced in the regions of α‐helices and β‐sheets, suggesting that GmSNAT3.1 and GmSNAT3.2 likely share functional parallels with ZmSNAT3.

### 2.5 Functional Validation of GmSNAT3.1 and GmSNAT3.2

**FIGURE 6:**
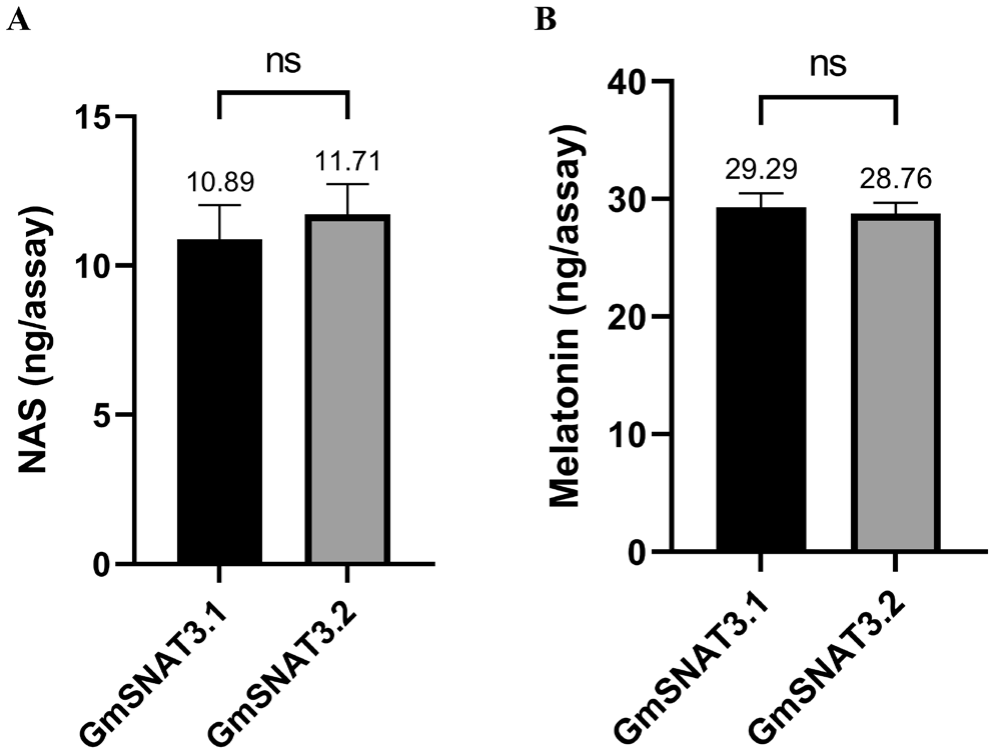
Enzymatic activity of the recombinant ZmSNAT1 and ZmSNAT3, with the substrates of serotonin (A) and 5‐MT (B).

To verify the SNAT enzyme activity of GmSNAT3.1 and GmSNAT3.2, the predicted chloroplast transit peptides (cTP) were removed, and the proteins were fused with a Maltose‐Binding Protein (MBP) tag to facilitate purification (designated as Δ51GmSNAT3.1 and Δ53GmSNAT3.2). In vitro enzymatic assays revealed that both Δ51GmSNAT3.1 and Δ53GmSNAT3.2 were capable of synthesizing N‐acetylserotonin (NAS) or melatonin when provided with serotonin or 5‐methoxytryptamine (5‐MT) as the substrate, respectively (Figure 6A,B). These results confirmed that *GmSNAT3.1* and *GmSNAT3.2* encoded functional SNAT enzymes. Notably, the catalytic activities of the two proteins were not significantly different, indicating that the observed variations in their amino acid sequences do not impair substrate binding or enzymatic efficiency. This suggested a high degree of functional conservation between the two proteins.

### 2.6 Tissue‐Specific Transcription of *GmSNAT3.1* and *GmSNAT3.2* and Their Response to Dehydration and Salt Stress

**FIGURE 7:**
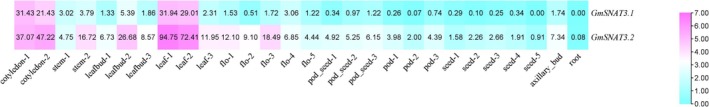
Transcription of GmSNAT3.1 and GmSNAT3.2 in different tissues of the soybean.

To investigate the tissue‐specific expression patterns of *GmSNAT3.1* and *GmSNAT3.2*, transcription levels were analyzed using data from the SoyOmics database. Both genes exhibited the highest expression in cotyledons and leaves, with moderate expression observed in stems, leaf buds, and flowers (ranging from 0.51 to 26.68). In contrast, their expression was minimal in pods and seeds (less than 10) (Figure 7). Notably, *GmSNAT3.2* showed higher transcriptional activity compared to *GmSNAT3.1* across most tissues, suggesting potential functional differences or regulatory divergence between the two genes. These findings highlighted the tissue‐specific roles of *GmSNAT3.1* and *GmSNAT3.2* in soybean development and stress responses.

**FIGURE 8:**
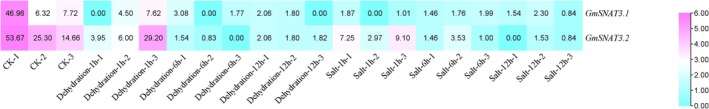
Transcription of GmSNAT3.1 and GmSNAT3.2 in soybean plants subjected to dehydration and salt stress.

Melatonin has been demonstrated to play a crucial role in plant responses to abiotic stresses. In this study, we investigated the expression patterns of *GmSNAT3.1* and *GmSNAT3.2*, key genes involved in melatonin biosynthesis, in soybean leaves under dehydration and salt stress conditions. Through comprehensive analysis of RNA‐seq data obtained from the GEO database, we observed significant downregulation of both *GmSNAT3.1* and *GmSNAT3.2* in the *Williams 82* cultivar when exposed to these stress conditions. These findings suggest that soybean plants may modulate their melatonin biosynthesis as an adaptive mechanism to cope with dehydration and salt stress, potentially through the regulation of these key biosynthetic genes (Figure 8). The coordinated downregulation of *GmSNAT3.1* and *GmSNAT3.2* under stress conditions indicated a complex regulatory network governing melatonin metabolism in response to environmental challenges in soybean plants.

### 2.7 The Protein–Protein Interaction and GO Analysis

**FIGURE 9:**
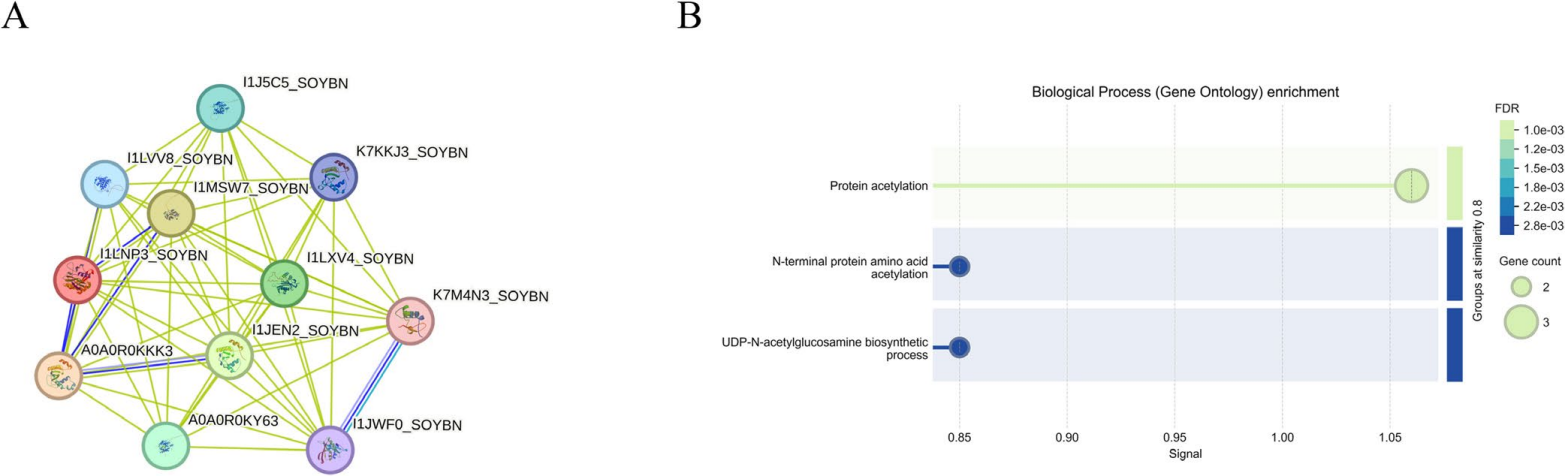
The protein–protein interaction (PPI) network and GO enrichment analysis based on the STRING database. (A) The STRING protein‐protein interaction network of GmSNAT3.1 with other soybean proteins (http://string‐db.org/). Colored lines between the proteins indicate the various types of interaction evidence. (B) GO enrichment analysis of the predicted proteins in the network.

The protein–protein interaction (PPI) network analysis using the STRING database identified potential interacting partners of GmSNAT3.1, including eight N‐acetyltransferase domain‐containing proteins and two glucosamine 6‐phosphate N‐acetyltransferases (Figure 9A). The predicted interaction profiles of GmSNAT3.2 exhibit high consistency with those of GmSNAT3.1, which can be attributed to their remarkably high sequence similarity. The results indicated that the proteins are at least partially biologically connected as a group. Gene Ontology (GO) enrichment analysis revealed that these proteins are significantly associated with protein acetylation, suggesting their potential roles in the acetylation of substrates in soybeans and post‐translational modification processes (Figure 9B).

### 2.8 Identification of SNAT3 Genes in Leguminosae Family

**FIGURE 10:**
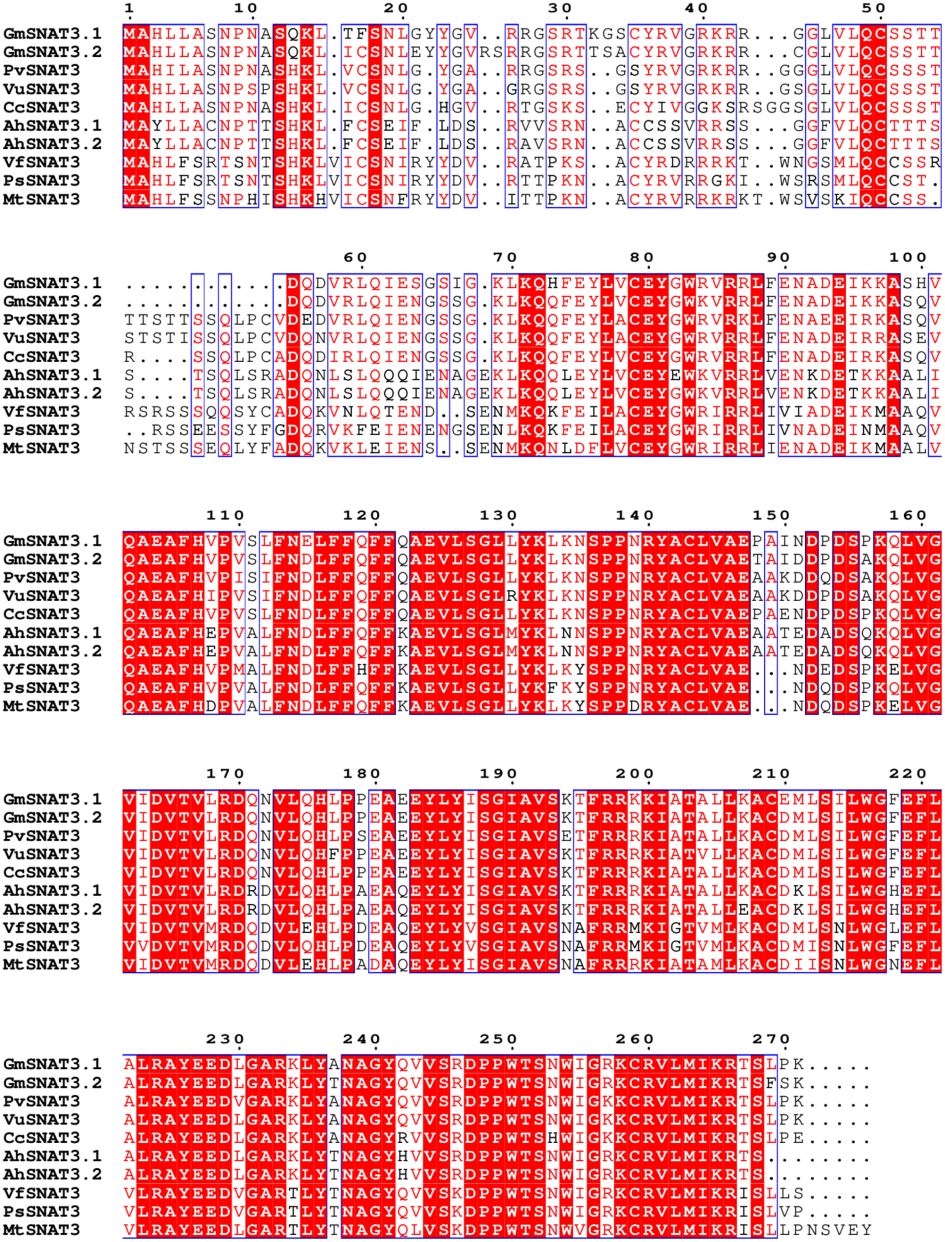
Multi‐sequence alignment of SNAT3 proteins in some plants.

Building upon the identification of *GmSNAT3.1* and *GmSNAT3.2* in soybean, we extended our investigation to identify SNAT3 homologs across other members of the Leguminosae family. Using the newly characterized soybean SNAT3 genes as reference sequences, we performed a comprehensive search for orthologous genes in related legume species. Multiple sequence alignment revealed the remarkable conservation in the amino acid sequences of these SNAT3 proteins, with observed variations primarily localized to the N‐terminal chloroplast transit peptide region (Figure 10). This high degree of sequence conservation, consistent with previous findings on other conserved enzyme systems such as peroxidases and lipase isoenzymes in legumes, strongly suggested that SNAT3 enzymes maintain conserved functional roles across different legume species. The evolutionary conservation of these genes underscored their fundamental importance in melatonin biosynthesis and related physiological processes within the Leguminosae family.

## 3 Discussion

Melatonin is a multifunctional molecule present in both animals and plants. The endogenous melatonin content plays a crucial role in plant responses to environmental changes (Kouser et al. 2025; Fahad et al. 2025). Regulating endogenous melatonin levels is more time‐ and energy‐efficient compared to exogenous applications. Given the significant role of melatonin in plant growth and development, as well as recent advances in cloning genes involved in its biosynthesis (Back et al. 2021; Zheng et al. 2021; Zhou et al. 2021; Kumar et al. 2022; Zhang et al. 2022; Chen, Wu, et al. 2023; Guo et al. 2024), it is imperative to continue this research to fully understand and potentially manipulate the melatonin synthesis pathway in plants. Melatonin primarily functions as a scavenger of free radicals in all organisms. Since plants produce reactive oxygen species (ROS) during aerobic metabolism, melatonin biosynthesis predominantly occurs in mitochondria and chloroplasts, as indicated by Back (2021). In this study, GNAT family members predicted to contain chloroplast transit peptides were identified. Through phylogenetic analysis, two additional GmSNAT genes, *GmSNAT3.1* and *GmSNAT3.2*, were identified, besides the already reported GmSNAT1 (Kumar et al. 2022). Notably, the tertiary structures of GmSNAT3.1 and GmSNAT3.2 exhibited high similarity to ZmSNAT3 from maize, suggesting functional conservation across plant species.

In vitro experiments have confirmed that GmSNAT3.1 and GmSNAT3.2 can utilize serotonin or 5‐methoxytryptamine (5‐MT) as substrates to produce N‐acetylserotonin (NAS) or melatonin, consistent with the characteristics of SNAT enzymes observed in other plant species. The enzymatic functionality of GmSNAT3.1 and GmSNAT3.2 highlighted their critical role in regulating melatonin levels in soybeans. Melatonin has been shown to enhance stress tolerance, promote root development, and improve seed germination in various plants (Arnao and Hernández‐Ruiz 2015; Zhang et al. 2013; Wei et al. 2015).

*GmSNAT3.1* and *GmSNAT3.2* exhibited the highest transcriptional activity in cotyledons and leaves, indicating their potential significance in photosynthetic tissues or early developmental stages. The elevated expression of melatonin in leaves aligned with its protective role in photosynthetic tissues, where reactive oxygen species (ROS) are commonly generated under stress conditions (Ahammed et al. 2024). This suggested that these genes may play a crucial role in maintaining melatonin levels in leaves. Additionally, *GmSNAT3.2* generally displayed higher expression levels than *GmSNAT3.1* across most tissues, implying it may have a more prominent functional role under normal conditions. Similar observations have been documented in other plant species, where multiple isoforms of melatonin biosynthesis genes exhibit differential expression patterns (Lee, Byeon, Lee, et al. 2014). Both *GmSNAT3.1* and *GmSNAT3.2* were downregulated under dehydration and salt stress conditions. A comparable phenomenon was observed in maize, where *ZmSNAT1* and *ZmSNAT3* were significantly downregulated under heat stress (Guo et al. 2024). However, this downregulation contrasted with findings in some other plant species, where melatonin biosynthesis genes are often upregulated under stress (Wang et al. 2017). The downregulation of these genes might be a mechanism to prevent excessive melatonin accumulation, which could have adverse effects under certain conditions (Wei et al. 2015). Therefore, the identification of GmSNAT3.1 and GmSNAT3.2 as key enzymes in melatonin biosynthesis has been recognized as potential targets for genetic engineering aimed at enhancing stress tolerance in soybeans.

The identification of interacting proteins within the N‐acetyltransferase family underscores the potential functional synergy between GmSNAT3.1 and GmSNAT3.2 and other acetyltransferases. Protein acetylation, as indicated by GO enrichment (Figure 9B), is a critical regulatory mechanism influencing protein stability, activity, and interactions (Glozak et al. 2005; Zhao et al. 2010). These findings align with previous studies demonstrating the importance of acetylation in metabolic regulation and stress responses (Hartl et al. 2011). However, the predicted interactions require experimental validation, such as co‐immunoprecipitation or yeast two‐hybrid assays, to confirm their biological relevance. Additionally, the functional implications of these interactions in specific physiological contexts, such as plant development or environmental adaptation, remain to be explored.

The high sequence and structural similarity among GmSNAT3.1, GmSNAT3.2, and ZmSNAT3 from maize, coupled with the evolutionary conservation of melatonin biosynthesis pathways across various plant species, suggested a conserved mechanism for melatonin production. The catalytic activities of GmSNAT3.1 and GmSNAT3.2 were remarkably similar, indicating that the amino acids responsible for maintaining enzyme activity are highly conserved. This highlighted both the evolutionary significance of these genes and their crucial role in plant adaptation to environmental stresses. Furthermore, the identification of SNAT3 homologs in several legume species confirmed the conservation of this pathway within the Leguminosae family.

## 4 Materials and Methods

### 4.1 Identification of Soybean GNAT Family Genes and SNAT3 in Selected Legume Plants

The soybean genome was downloaded from Ensembl Plants (Glycine_max—Ensembl Genomes 60). We utilized the Hidden Markov Model of the GNAT domain (PF00583) as a query to search for proteins within the soybean genome (Yu et al. 2019), setting the threshold at e < 1e‐5. The genomes of other legume plants used in this study were also downloaded from Ensemble Plants. Identification of SNAT3 candidate genes in selected legume plants was analyzed by the module “Find Best Homology” in TBTools v2.112 (Chen, Zhang, et al. 2023).

### 4.2 Chromosome Position and Domain Analysis

Chromosome position and domain analysis of GmGNAT family members was performed by TBtools v2.112 (Chen, Wu, et al. 2023). The domain analysis was performed in InterPro (InterProScan–InterPro (ebi.ac.uk)) and the resulting picture was displayed and generated by.

TBtools v2.112 (Chen, Zhang, et al. 2023).

### 4.3 The Sequence Alignments and Phylogenetic Analysis of GmGNATs

The protein sequences of GmGNAT and newly identified ZmSNAT3 in maize (Guo et al. 2024) were integrated with nucleotide sequences for comprehensive phylogenetic analysis. Multiple sequence alignments were performed using MAFFT version 7 (MAFFT alignment and NJ/UPGMA phylogeny (cbrc.jp)) and the phylogenetic tree was constructed by the IQ‐TREE web server (IQTREE Web Server: Fast and accurate phylogenetic trees under maximum likelihood (univie.ac.at)) with the default parameter.

### 4.4 Comparison and Visualization of Protein Tertiary Structures

The tertiary structures of GmSNATs and ZmSNAT3 were downloaded from AlphaFold2 (AlphaFold Protein Structure Database (ebi.ac.uk)). The tertiary structure alignment was performed, and the RMSD was calculated by Pymol 2.5.2.

### 4.5 Production of GmSNAT Recombinant Proteins in *Escherichia coli*

Both GmSNAT3.1 and GmSNAT3.2 (not GmSNAT3.1 repeated) harbor a short chloroplast transit peptide (cTP) sequence, as predicted by ChloroP (Emanuelsson et al. 1999). To exclude the cTP sequence from the recombinant proteins, PCR primers were specifically designed to amplify the coding sequences of GmSNAT3.1 or GmSNAT3.2 (excluding the cTP portion) from the soybean cultivar *Williams 82* (Table S1). The PCR amplicons thus amplified from ZmSNAT1 and ZmSNAT3 cDNA were then inserted into the pMAL‐C6T vector linearized by AlwnI. The recombinant vector was subsequently transformed into *E. coli* BL21 (DE3) (purchased from TransGen Biotech Co. Ltd., Beijing, China). To produce recombinant GmSNATs protein, the transformed *E. coli* were inoculated onto Luria‐Bertani (LB) agar medium containing 100 mg/L ampicillin (components include 10 g/L tryptone, 5 g/L yeast extract, 10 g/L sodium chloride, and 15 g/L agar), and a single colony was picked and transferred into 5 mL of LB broth containing 100 mg/L ampicillin (components include 10 g/L tryptone, 5 g/L yeast extract, 10 g/L sodium chloride). The culture was then incubated overnight at 37°C. 3 mL of the overnight culture was inoculated into 300 mL of LB medium containing 100 mg/L ampicillin until the optical density (OD600) of the *E. coli* reached 0.6–0.8. Subsequently, 200 μM of isopropyl‐β‐D‐thiogalactoside (IPTG; Sigma, St. Louis, MO, USA) was added, and the culture was incubated at 16°C with shaking at 160 rpm for 20 h. After lysing the *E. coli* cells using a high‐pressure homogenizer, the following purification steps using amylose resin were performed according to the manufacturer's instructions (NEB, Beijing, China). The purified proteins were concentrated using Amicon ultra‐4 centrifugal filters (Merck Millipore, Carrigtwohill, Ireland) and finally dissolved in PBS buffer at pH 8.0.

### 4.6 Verification of SNAT Enzyme Activity

Purified recombinant SNATs were utilized for the enzymatic activity assay. The entire reaction was conducted in a total volume of 100 μL, which comprised 0.5 mM of 5‐hydroxytryptamine (or 5‐MT) as the substrate and 0.5 mM of acetyl‐CoA. The reaction was carried out in PBS buffer with a pH value of 8.0. Subsequently, the mixture was incubated at 45°C for 3 h, and the reaction was terminated by the addition of 200 μL of methanol (MeOH). The reaction buffers were then centrifuged at 10,000 rpm for 2 min, and the supernatants were filtered through a PTFE membrane (0.22 μm). A 5 μL aliquot of the filtered supernatant was used for the determination of N‐acetylserotonin (NAS) or melatonin on an HPLC equipped with a fluorescence detector system (LC‐20, Shimadzu). The separation of all chemical substances was performed at a flow rate of 1 mL/min using an Ultimate XB‐C18 chromatographic column (4.6 × 150 mm; Welch, Shanghai, China) with isocratic elution. The elution solution was a mixture of 30% methanol and 0.1% formic acid, and the elution lasted for 20 min. The detection wavelengths for NAS and melatonin were set at an excitation wavelength of 280 nm and an emission wavelength of 348 nm, respectively. All measurements were reproduced in triplicate to ensure the accuracy and reliability of the results. The concentration of the recombinant proteins was determined by the Bradford method using a protein assay dye (Bio‐Rad, Hercules, CA, USA).

### 4.7 Source of Transcriptome and Visualization of Transcription Profile

For analysis of the tissue‐specific expressions of GmSNAT3.1 and GmSNAT3.2, the revelation of the profile was performed in the SoyOmics database (SoyOmics–CNCB‐NGDC). The dataset of dehydration and salt stresses was downloaded from the GEO database with the accession GSE57252. The heatmap was generated by TBtools v2.112 (Chen, Wu, et al. 2023).

### 4.8 The Protein–Protein Interaction and GO Analysis

The protein–protein interaction (PPI) network construction and Gene Ontology (GO) enrichment analysis were conducted using the STRING database. Statistical analysis revealed a highly significant PPI enrichment (*p*‐value < 1e^−16^). GO enrichment analysis demonstrated significant functional associations, with a false discovery rate (FDR) of less than 0.01.

### 4.9 Statistical Analysis

For statistical analysis, the Student's *t*‐test method was employed.

## 5 Conclusions

In conclusion, this study has identified and functionally characterized two novel GmSNAT genes—*GmSNAT3.1* and *GmSNAT3.2*—in soybeans. These genes encode SNAT enzymes capable of converting serotonin or 5‐MT into NAS or melatonin. RNA‐seq data revealed that transcription levels of *GmSNAT3.1* and *GmSNAT3.2* were downregulated under conditions of dehydration and salt stress. Additionally, we have identified SNAT3 proteins in several other leguminous plants as well. These findings not only establish a solid foundation for future research on the physiological functions of melatonin in soybeans and other legume species but also hold significant potential for enhancing crop resilience and productivity amid various environmental challenges.

## Author Contributions

**Jiajia Zhang:** data curation (lead), formal analysis (lead), investigation (equal), validation (lead), writing – original draft (lead). **Haopeng Li:** formal analysis (supporting), investigation (equal), methodology (lead), validation (supporting), visualization (equal). **Hongfei Ma:** investigation (supporting), visualization (equal), writing – original draft (supporting). **Wei Yan:** methodology (supporting), software (lead). **Jianbo Zhu:** project administration (lead), resources (lead), supervision (lead), writing – review and editing (lead).

## Conflicts of Interest

The authors declare no conflicts of interest.

## Supporting information


Data S1.

Data S2.

## Data Availability

The data that support the findings of this study are available on request from the corresponding author.
