# Supplementary material for: Genome‐Wide Identification and Functional Characterization of New Serotonin N‐Acetyltransferases in Soybean
Source: Food Sci Nutr. 2025 Apr 6;13(4):e70147. doi: 10.1002/fsn3.70147 (PMC11972975; doi:10.1002/fsn3.70147)
Supplement: Supplementary file 2 — Data S2. [file FSN3-13-e70147-s002.docx]

**Table S1** The primers used in this study for constructing the vector.

| Primers | Sequence (5’→3) |
| --- | --- |
| GmSNAT3.1-MBP-F | ggggagaacctgtacttccagtctAGCACAACTGATCAGGATGTGAGG |
| GmSNAT3.1-MBP-R | gatatcgcggccgcccatcagcatCTATTTGGGCAAACTAGTTCTTTTGA |
| GmSNAT3.2-MBP-F | ggggagaacctgtacttccagtctAGCACAACTGATCAGGATGTGAGG |
| GmSNAT3.2-MBP-R | gatatcgcggccgcccatcagcatCTATTTGGAGAAACTAGTTCTTTTGATCA |

Note: Lowercase letters are homology arms.


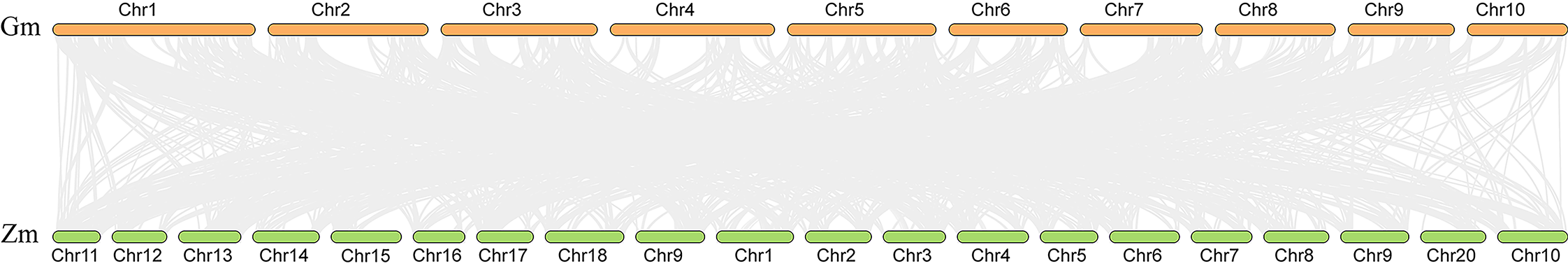


**Figure S1** The synteny analysis of soybean and maize genome

The species abbreviations are denoted as follows: Gm for *Glycine max* (soybean) and Zm for *Zea mays* (maize). Gray lines represent syntenic blocks between the corresponding chromosomal regions.
